# Functional analysis of granulocyte and monocyte subpopulations in neonates

**DOI:** 10.1186/s40348-019-0092-y

**Published:** 2019-11-28

**Authors:** Ines Hegge, Ferry Niepel, Anja Lange, Antje Vogelgesang, Matthias Heckmann, Johanna Ruhnau

**Affiliations:** 1 Department of Neonatology and Pediatric Intensive Care, University Medicine Greifswald, Greifswald, Germany; 2 Department of Neurology, University Medicine Greifswald, F.-Sauerbruch-Str., 17475 Greifswald, Germany

**Keywords:** Neonate, Subpopulations, Innate immunity, ROS

## Abstract

**Background:**

Neonate immune cell functions lack full protection against pathogens. This could be either defect or protective mechanism against overshooting proinflammatory immune responses.

We here analysed the function of classical, pro- and anti-inflammatory monocytes and granulocytes from neonates in comparison with adults to investigate if suppressed functions of subpopulations are causative for the unique neonatal immune status. Therefore, reactive oxygen species (ROS) and surface activation markers were quantified in subpopulations.

**Methods:**

In a prospective, longitudinal study granulocyte and monocyte subpopulations were analysed in healthy term infants (> 37 week; *n* = 13) in comparison with healthy young adults (*n* = 11). Percentage (%) of cells expressing surface marker (HLA-DR, CD11b, CD62L, CD32, Toll-Like-Receptor-2) and expression per cell, determined by mean fluorescence intensity (MFI), were measured by flow cytometry. ROS production was induced by fMLP, PMA and *E. coli* in term neonates (> 37 week; *n* = 13).

**Results:**

Classical granulocytes were down- and proinflammatory granulocytes upregulated in neonates compared with adults. Percentage of TLR-2 expressing granulocytes was increased in neonates. Granulocytic ROS production depended on stimulation. The percentage of anti-inflammatory monocytes was increased, while classical monocytes were reduced in neonates. HLA-DR (%, MFI) showed reduction for all monocyte subpopulations, while CD32, CD11b, CD62L and TLR-2 were differently regulated in comparison with adults.

**Conclusions:**

Differentially regulated granulocyte and monocyte subpopulations indicate a unique state of neonatal immunity to fight infections and prevent dysregulation. Further studies are needed to investigate the role of reduced granulocytic ROS formation and reduced monocytic HLA-DR in active disease.

## Background

As the adaptive immunity of newborns is mostly naive and immune responses will take longer to develop as compared with adults, the innate immune response, mainly carried out by neutrophils and monocytes, is of major importance for early-life pathogen defence [1]. But newborn neutrophils show characteristics of impaired bacterial defence function like reduced expression of Toll-like receptor (TLR) 2 [2] and reduced formation of neutrophil extracellular traps [3]—important mechanisms for recognition and trapping of pathogens. Furthermore, monocytes express less HLA-DR which is known to be a marker of immunosuppression and a predictor of sepsis development in neonates [4]. Monocytes are also impaired in uptake of bacteria by phagocytosis [5].

Recently Brook et al. stated that the partly lowered antibacterial function of innate immune cells like neutrophils and monocytes is rather a mechanism of protection from overshooting proinflammatory immune responses than a pathologic defect [6]. A broad range of pro-inflammatory plasma proteins is upregulated in septic neonates in comparison with healthy controls, indicating an overwhelming and consuming proinflammatory immune response against infection in early life [7].

Granulocytes have been differentiated in three different main subpopulations by CD16, a Fc-receptor, and CD62L, a L-Selectin which is important for cell adhesion [8–10]. The largest granulocyte subset within the peripheral blood, referred to as “classical granulocytes,” expresses high levels of CD16 and to some degree CD62L (CD16^+^CD62L^+^), proinflammatory granulocytes are defined as CD16^dim^CD62L^+^ and anti-inflammatory granulocytes as CD16^+^CD62L^−^. To date, no data about the different regulation of granulocyte subpopulations in neonates exist. Similar to granulocytes, monocytes can be distinguished in three different subpopulations in regard to their expression of CD14 and CD16: (i) classical monocytes (CD14^+^CD16^−^); (ii) anti-inflammatory (CD14^+^CD16^+^); and (iii) proinflammatory monocytes (CD14^dim^CD16^+^).

An altered composition of granulocyte or monocyte subsets might be part of the unique immune system configuration after birth which prevents accelerating immune responses. Therefore, we analysed granulocytes and monocytes in term neonates to gain a better understanding of the regulation of their subpopulations in comparison with young adults. This knowledge will allow future studies to investigate the role of these subpopulations for increased infection rates in term or preterm neonates.

The Fc-receptor CD32 mediates multiple cell-type specific functions including phagocytosis or release of inflammatory mediators, which are important functions of innate immune cells [11]. CD11b plays a critical role in pathogen recognition. Its downstream signalling initiates immune responses that ultimately link to the generation of adaptive immunity [12]. Since alterations of activation markers could be a part of the optimal balance between immune resistance and response TLR-2, HLA-DR, CD32, CD11b and CD62L were analysed on monocyte and granulocyte subpopulations.

The production of reactive oxygen species (ROS) is one of the best analysed anti-bacterial mechanisms of neonatal neutrophils. Nevertheless, it is partly discussed controversially [13, 14]. ROS production within respiratory burst of neonates was reported to be as effective as the one of adults [15]. In contrast stressed neonatal neutrophils showed a significantly elevated response when compared with those from adult controls [16]. Since these data lack an analysis of granulocyte subpopulations we here analysed the three subpopulations in regard to their production of ROS as determined by the conversion of dihydrorhodamine (DHR) into rhodamine.

## Methods

### Study population

Healthy term neonates were recruited between July 2016 to March 2017 and May 2018 to September 2018 at the University Medicine Greifswald. Healthy young adults served as controls.

Monocyte and granulocyte subpopulations were analysed in 11 healthy term neonates (mean gestational age = 39 weeks + 2 days (SD, 1 weeks + 2 days); male = 7, female = 4; birth mode: spontaneous = 1; primary caesarean section = 9; secondary caesarean section = 1) and 10 young healthy adults (mean age = 23.5 years (SD = 4.9 years), male = 2, female = 8).

For ROS production experiments samples of 13 healthy term neonates (mean gestational age, 38 weeks + 4 days (SD, 0 weeks + 6 days); male = 5, female = 8; birth mode: spontaneous = 5; primary caesarean section = 8; secondary caesarean section = 0) and 11 young healthy adults (mean age, 25 years (SD, 6.4 years); male = 5, female = 6) were used.

Exclusion criteria were severe congenital malformations, chromosomal aberrations, perinatal infection or chorioamnionitis and lack of written consent (see Additional file 1: Table S1 for details).

### Monocyte and granulocyte subtypes

EDTA cord blood was sampled at birth and processed within 2 h to examine neonatal monocytes and granulocytes. After a red blood cell lysis using ACK lysing buffer (155 mM NH_4_Cl, 10 mM KHCO_3_, 0.1 mM EDTA) to induce cell swelling and rupture of membranes, cells were stained by Zombie NIR™ Fixable Viability Kit (BioLegend™) on ice for 15 min to distinguish dead and alive cells, followed by a second staining with the different cell surface antibodies for 10 min on ice. Cells were fixed in 1% paraformaldehyde (PFA). Immune cell subpopulations of monocytes and granulocytes were analysed by flow cytometry (BD LSR II) as defined by anti-HLA-DR Alexa Flour 488, anti-CD11b Brilliant Violet 421, anti-CD14 PerCP/Cy5.5, anti-CD16 Brilliant Violet 650, anti-CD62L PE-Cy7, anti-TLR-2 Alexa 647 and anti-CD32-PE (BioLegend). The results were evaluated using FlowJo Software 7.6.5 (Tree Star Inc., Ashland, OR, USA). Results are shown as percentages of granulocytes, monocytes or their subpopulations. To display the expression of activation markers, mean fluorescence intensity (MFI) was used. For the differentiation of monocytes and granulocyte subpopulation as well as activation marker, fluorescence minus one controls (FMO) were used. CD14^dim^ monocyte and CD16^dim^ neutrophil population was distinguished by gating the 25th percentile of main monocyte and neutrophil population, respectively (Additional file 2: Figure S1A/B).

### ROS production

ROS production was quantified by flow cytometry using the Phagoburst Kit (Glycotope Biotechnology GmbH) according to manufacturer’s instructions. Briefly, heparinized cord blood was incubated on ice with anti-CD14 APC/Cy7, anti-CD16 APC and anti-CD62L Brilliant Violet 421 antibodies (Biolegend). For detailed gating strategy see Additional file 3: Figure S2. Cells either remained unstimulated or were incubated with unlabeled opsonized *E. coli* (0.9–1.8 × 10^8^/ml), phorbol 12-myristate 13-acetate (PMA) (0.74 μM), or N-formyl-methionyl-leucyl-phenylalanine (fMLP) (0.45 μM) as stimulants for 10 min at 37 °C; subsequently, DHR was added for 10 min which—by ROS-dependent conversion into rhodamine 123—allowed the quantification of reactive oxidants and determination of the percentage of phagocytes that produced ROS. The ROS production per cell was quantified by MFI. Kit-included DNA-Dye was used after red blood cell lysis using PFA-containing BD FACS™ Lysing Solution to differentiate between *E. coli* and cells. The flow cytometry results were evaluated with FlowJo Software 10.3 (Tree Star Inc., Ashland, OR). Gating of subpopulation was done as described above (Additional file 2: Figure S1 A/B; Additional file 3: Figure S2).

### Statistical analysis

All data sets were tested for adherence to the Gaussian distribution with the Kolmogorov-Smirnov test. Since some of the data failed the normality test we used non-parametric testing throughout. The Kruskal-Wallis test or Friedman test with Dunn’s multiple comparison test as a post-test or the Mann-Whitney test were used as appropriate. GraphPad-PRISM 5.0 (GraphPad Software Inc., San Diego, CA, USA) was used for all analyses. A *p* value ≤ 0.05 was regarded as significant.

## Results

### Differently regulated granulocyte subpopulations

**Fig. 1:**
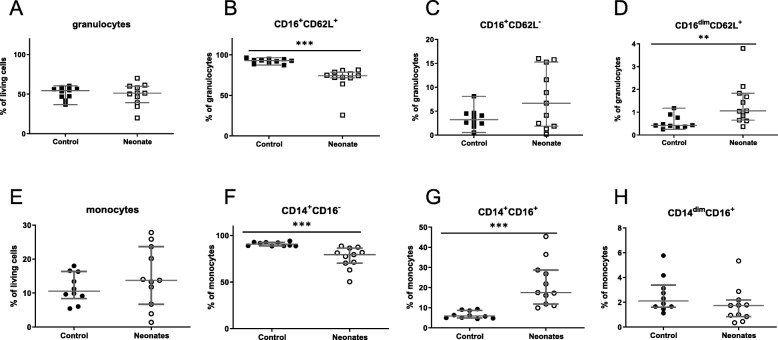
Granulocyte and monocyte subpopulations. Percentage of granulocyte (squares) and monocyte subpopulation (dots) is shown for term neonates (white) and healthy young adults (black). Apart from total granulocytes (in squares) (**a**), granulocyte subpopulations were defined via the expression of CD16 and CD62L in three subpopulations: classical granulocytes (CD16^+^CD62L^+^) (**b**); immunosuppressive granulocytes (CD16^+^CD62L^−^) (**c**); inflammatory granulocytes (CD16^dim^CD62L^+^) (**d**). Total monocytes are shown in **e**. In addition monocytes (dots) were also distinguished in three subpopulations: anti-inflammatory (CD14^+^CD16^+^) (**f**), the classical monocytes (CD14^+^CD16^−^) (**g**); and proinflammatory monocytes (CD14^dim^CD16^+^) (**h**).**p < 0.01; ***p < 0.005. Scattered plot with medians and interquartile

Compared with adult controls neonates showed a reduced percentage of classical granulocytes CD16^+^CD62L^+^cells while proinflammatory CD16^dim^CD62L^+^cells were increased. Anti-inflammatory CD16^+^CD62L^−^ cells were not altered in comparison with controls (Fig. 1a–d).

### Activation marker of granulocyte and their subpopulations

To analyse the activation profile of granulocytes and their subpopulations in comparison with controls, we analysed the percentage of CD11b, CD32 and TLR-2 expressing cells as well as the amount of activation marker per cell by MFI.

**Fig. 2:**
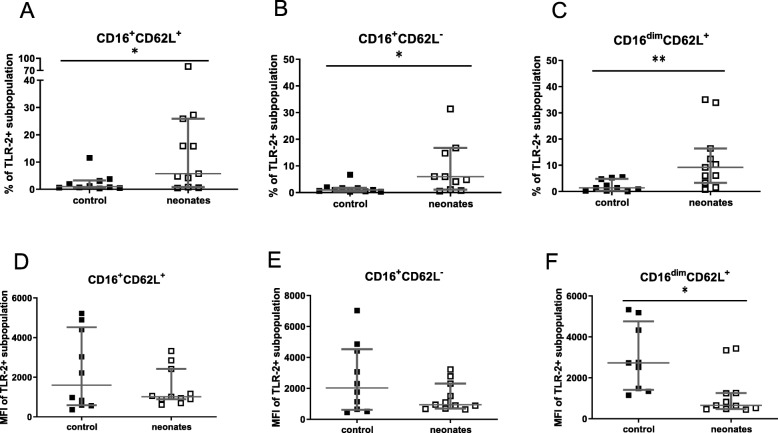
TLR-2 as activation marker on granulocytes and granulocyte subpopulations. Neonates (white squares) were compared with healthy controls (black squares). Percentage (%) (**a**–**c**) TLR-2 on the surface of cells and expression of TLR-2 as mean fluorescence intensity (MFI) (D-F) is shown for different granulocyte subset. In (**a**) and (**d**) classical granulocytes (CD16^+^CD62L^+^); in (**b**) and (**e**) immunosuppressive granulocytes (CD16^+^CD62L^−^) and in (**c**) and (**f**) inflammatory granulocytes (CD16^dim^CD62L^+^) are shown. *p < 0.05; **p < 0.01. Scattered plot with medians and interquartile

Reduced percentages of CD11b positive granulocytes and CD32 positive granulocytes were detected in neonates in comparison with controls. Neonates showed a higher percentage of TLR-2 on all granulocyte subpopulations compared with controls (Table 1). The amount of TLR-2 expression on proinflammatory CD16^dim^CD62L^+^ granulocytes was decreased in neonatal cord blood (Fig. 2).

**Table 1:** Summary of all observed marker on granulocyte and monocytes and their subpopulations

	Marker	Change (compared with adult)	Adult (median (Min–Max))	Neonate (median (Min–Max))	*p* value (Mann-Whitney test)
Granulocytes	CD11b+ (%)	↓	98.94 (97.83–99.78)	95.27 (29.87–97.73)	0.0001
	CD11b+ (MFI)	-	3423 (2274–6123)	2936 (1847–5151)	0.5974
	CD16+ (%)	↓	98.05 (93.91–99.32)	89.59 (28.21–96.12)	0.0002
	CD16+ (MFI)	↓	58.687 (40.735–76.909)	26.939 (26.309–36.276)	0.0001
	CD32+ (%)	↓	99.53 (98.11–99.85)	97.14 (32.18–99.41)	0.0017
	CD32+ (MFI)	-	16.466 (12.504–27.052)	19.779 (11.886–24.551)	0.3418
	CD62L+ (%)	↓	96.55 (93.15–99.34)	85.07 (32.05–92.36)	0.0001
	CD62L+ (MFI)	↓	43.841 (21.105–64.313)	19.172 (12.638–31.370)	0.0028
	TLR2+ (%)	↑	2.9 (0.6–10.98)	8.36 (3.32–72.73)	0.0035
	TLR2+ (MFI)	-	1506 (190.9–5427)	778.7 (365.3–3335)	0.8053
Granulocytes subsets	CD16+ CD62L+	↓	93.01 (87.46–96.55)	74.37 (25.78–81.35)	0.0001
	CD16+ CD62L−	-	3.24 (0.54–8.1)	6.68 (0.21–16.0)	0.2453
	CD16dim CD62L+	↑	0.425 (0.25–1.18)	1.06 (0.37–3.8)	0.0092
CD16+CD62L+ (classical)	CD11b+ (%)	-	100.0 (98.67–100)	99.40 (97.76–100)	0.0545
	CD11b+ (MFI)	-	3439 (2284–6139)	2978 (1880–5259)	0.5974
	CD32+ (%)	-	100.0 (100.0–100.0)	100.0 (99.97–100.0)	-
	CD32+ (MFI)	-	16.655 (12.794–27.373)	20.943 (13.267–27.917)	0.098
	TLR2+ (%)	↑	0.985 (0.32–11.52)	5.75 (0.46–79.95)	0.0346
	TLR2+ (MFI)	-	1598 (360.3–5217)	1013 (613.4–3321)	0.8603
CD16+CD62L- (anti-inflammatory)	CD11b+ (%)	-	98.43 (94.62–99.85)	98.17 (92.92–99.48)	0.3787
	CD11b+ (MFI)	-	3332 (2040–5834)	2753 (1686–3902)	0.3418
	CD32+ (%)	-	99.99 (99.61–100.0)	99.96 (54.96–100.0)	0.0587
	CD32+ (MFI)	-	15.244 (11.030–26.700)	19.774 (13.674–27.184)	0.0726
	TLR2+ (%)	↑	0.98 (0.27–6.67)	5.97 (0.57–81.36)	0.0183
	TLR2+ (MFI)	-	2028 (443.6–7026)	941.5 (634.4–3216)	0.4181
CD16dimCD62L+ (pro-inflammatory)	CD11b+ (%)	-	98.46 (81.77–100)	97.05 (82.95–99.86)	0.5495
	CD11b+ (MFI)	-	2457 (1516–5741)	2731 (2171–4653)	0.3787
	CD32+ (%)	-	100 (98.5–100.0)	99.97 (99.36–100.0)	0.9697
	CD32+ (MFI)	-	8236 (5792–16.004)	7168 (4230–10.209)	0.5035
	TLR2+ (%)	↑	1.4 (0.0–5.5)	9.18 (0.73–35.08)	0.0043
	TLR2+ (MFI)	↓	2625 (0.00–5333)	657.8 (446.7–3439)	0.0378
Monocytes	CD11b+ (%)	↓	99.51 (98.50–99.96)	98.64 (95.76–99.33)	0.0137
	CD11b+ (MFI)	-	2694 (1256–6598)	2093 (993.4–4453)	0.1489
	CD16+ (%)	↑	9.33 (5.91–11.38)	21.26 (10.97–49.66)	0.0003
	CD16+ (MFI)	↓	12.126 (5745–19.915)	3684 (1817–5825)	0.0002
	CD32+ (%)	-	99.93 (99.04–99.98)	99.65 (92.43–99.82)	0.0724
	CD32+ (MFI)	-	22.733 (12.835–31.849)	19.335 (13.609–30.177)	0.5035
	CD62L (%)	↓	94.24 (55.77–96.31)	80.15 (27.67–96.15)	0.0083
	CD62L+ (MFI)	↓	28.574 (15.121–61.757)	11.974 (5130–25.963)	0.0011
	HLA-DR+ (%)	↓	99.51 (97.14–99.83)	94.46 (85.72–97.56)	0.0002
	HLA-DR+ (MFI)	↓	5050 (3839–5678)	2008 (1135–4030)	0.0002
	TLR2+ (%)	↓	99.07 (95.67–99.83)	97.27 (92.02–99.30)	0.0317
	TLR2+ (MFI)	-	3693 (2511–5553)	3166 (1271–5754)	0.1131
Monocyte subsets	CD14+ CD16+	↑	5.925 (4.47–9.33)	17.52 (9.89–45.38)	0.0001
	CD14+CD16-	↓	90.59 (88.62–94.09)	79.4 (50.34–88.55)	0.0001
	CD14dim CD16+	-	2.1 (1.12–5.77)	1.74 (0.35–5.34)	0.1299
CD14+CD16- (classical)	CD11b+ (%)	↓	99.77 (98.59–99.98)	98.26 (94.89–99.29)	0.0006
	CD11b+ (MFI)	-	2709 (1250–6706)	2025 (914.2–4189)	0.13
	CD32+ (%)	↓	99.94 (99.27–99.99)	99.67 (93.55–99.8)	0.0183
	CD32+ (MFI)	-	22.650 (12.310–31.390)	18.040 (12.880–27.480)	0.2453
	CD62L+ (%)	↓	96.81 (57.66–98.74)	82.53 (26.2–98.1)	0.0054
	CD62L+ (MFI)	↓	29.690 (15.240–64.070)	12.150 (5195–28.160)	0.0014
	HLA-DR+ (%)	↓	99.57 (98.29–99.87)	95.59 (88.84–99.24)	0.0003
	HLA-DR+ (MFI)	↓	4260 (3050–4736)	1833 (907.4–2658)	0.0001
	TLR2+ (%)	↓	99.69 (97.23–99.96)	97.89 (94.51–99.29)	0.0151
	TLR2+ (MFI)	-	3671 (2465–5485)	2944 (1225–5206)	0.0845
CD14+CD16+ (anti-inflammatory)	CD11b+ (%)	-	99.85 (97.72–100.0)	99.92 (98.06–100.0)	0.5714
	CD11b+ (MFI)	-	3009 (1441–6496)	2643 (1357–5016)	0.4181
	CD32+ (%)	-	99.93 (96.42–100.0)	99.77 (90.23–99.94)	0.4165
	CD32+ (MFI)	-	24.030 (14.400–40.560)	22.930 (16.130–34.550)	0.6985
	CD62L+ (%)	-	74.74 (35.97–86.96)	75.91 (42.13–95.42)	0.5973
	CD62L+ (MFI)	↓	16.240 (11.660–23.330)	12.150 (4641–20.090)	0.0221
	HLA-DR+ (%)	↓	99.16 (87.21–100.0)	93.53 (64.45–96.81)	0.0378
	HLA-DR+ (MFI)	↓	14.880 (10.390–19.610)	3219 (2243–7424)	0.0001
	TLR2+ (%)	-	98.54 (82.51–99.77)	95.53 (87.48–99.86)	0.5495
	TLR2+ (MFI)	-	4893 (3280–6613)	3546 (1492–6474)	0.0528
CD14^dim^CD16+ (pro-inflammatory)	CD11b+ (%)	-	98.17 (89.87–99.83)	96.82 (86.89–98.53)	0.3418
	CD11b+ (MFI)	-	1523 (803.7–3416)	1250 (1027–2207)	0.5974
	CD32+ (%)	-	98.87 (85.65–100.0)	96.11 (83.81–100.0)	0.09
	CD32+ (MFI)	-	15.860 (10.070–34.790)	14.650 (11.360–22.380)	0.2178
	CD62L+ (%)	-	32.15 (13.08–44.67)	34.89 (5.3–53.33)	0.8053
	CD62L+ (MFI)	-	9512 (4503–31.870)	16.650 (7670–25.480)	0.1131
	HLA-DR+ (%)	↓	92.48 (75.53–98.71)	63.51 (46.67–95.24)	0.0151
	HLA-DR+ (MFI)	↓	12.550 (7028–15.170)	8144 (3757–11.960)	0.0017
	TLR2+ (%)	-	84.39 (61.21–97.33)	76.67 (41.11–91.01)	0.1697
	TLR2+ (MFI)	-	3529 (2709–5068)	3104 (1390–4592)	0.13

### ROS production of granulocyte subpopulations

**Fig. 3:**
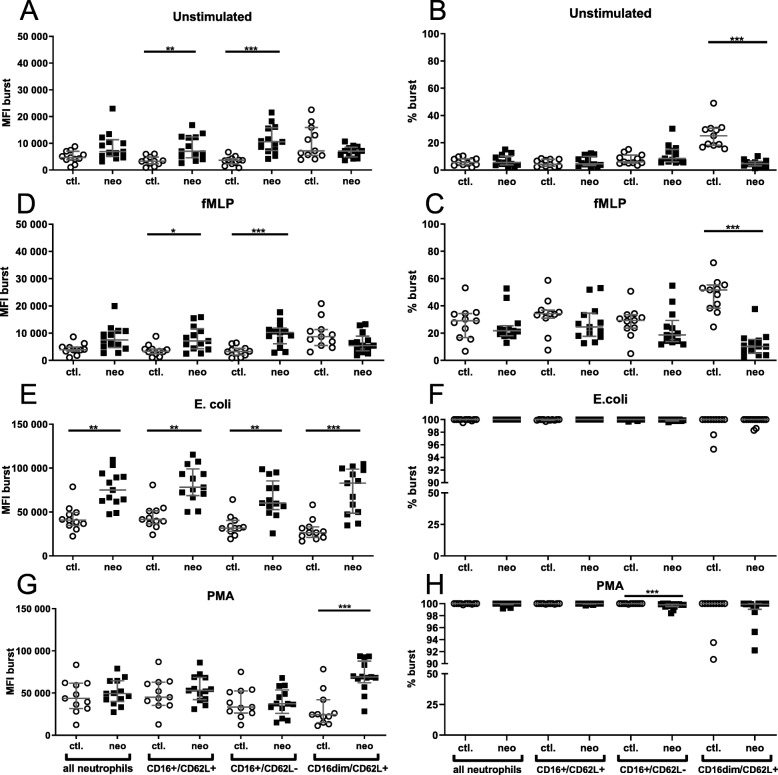
Oxidative burst of granulocyte subpopulations. Granulocyte and granulocyte subpopulations (CD16^+^CD62L^+^, CD16^+^CD62L^−^, CD16^dim^CD62L^+^) were analysed between healthy neonates (black squares) and healthy young adults (white dots). Oxidative burst was measured by mean fluorescence of cells (MFI) (**a**, **d**, **e**, **g**) as the production per cell as well as the percentage of cells producing ROS (%) (**b**, **c**, **f**, **h**). Cells were stimulated with fMLP (**d**, **c**), *E. coli* (**e**, **f**), PMA (**g**, **h**) or were left unstimulated (A, B). *p < 0.05; **p < 0.01; ***p < 0.005. Scattered plot with medians and interquartile

Percentage of ROS-producing granulocyte subpopulation and ROS amount per cell (MFI) was quantified (Fig. 3). Neonates showed enhanced ROS amount per cell for classical CD16^+^CD62L^+^ and anti-inflammatory CD16^+^CD62L^−^ subpopulation in unstimulated and fMLP-stimulated cells. In addition, ROS amount per cell was upregulated in proinflammatory CD16^dim^CD62L^+^cells after PMA stimulation. In *E. coli–*stimulated samples all neonatal granulocytes as well as their subpopulations showed a higher ROS production per cell in comparison with controls. A lower percentage of ROS-producing proinflammatory CD16^dim^CD62L^+^cells was measured for unstimulated and fMLP-stimulated cells in neonates. PMA stimulation induced a reduced percentage of ROS-producing anti-inflammatory CD16^+^CD62L^−^. An explorative subanalysis of sex showed no significant differences (*p* ≥ 0.9999; data not shown).

### Differently regulated monocyte subpopulations

Monocyte subpopulations were differentially regulated in neonates compared with controls. Classical CD14^+^CD16^+^ cells were enhanced in neonatal cord blood in comparison with lower number of anti-inflammatory CD14^+^CD16^−^ cells and unaltered proinflammatory CD14^dim^CD16^+^ cells (Fig. 1e–h).

### Activation marker of monocytes and their subpopulations

To analyse the activation profile of monocytes in comparison with healthy controls, percentages of HLA-DR, CD11b, CD62L and TLR-2 bearing cells as well as the amount per cell (MFI) were analysed.

Percentage of CD11b, CD62L, HLA-DR and TLR-2 bearing monocytes was reduced in neonatal compared with adult monocytes. The amount of CD62L and HLA-DR was significantly decreased on neonatal monocytes (Table 1).

**Fig. 4:**
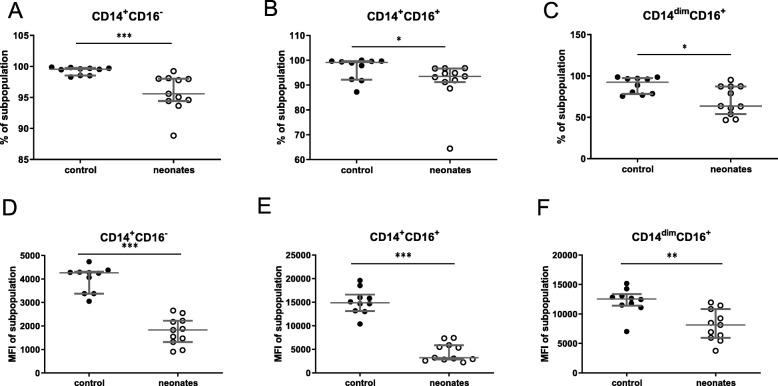
HLA-DR as activation marker on monocytes and monocyte subpopulations. Neonates (white dots) were compared with healthy controls (black dots). Percentage (%) (**a**–**c**) HLA-DR on the surface of cells and expression of HLA-DR as mean fluorescence intensity (MFI) (D-F) is shown for different monocyte subset. In (**a**) and (**d**) anti-inflammatory (CD14^+^CD16^+^); in (**b**) and (**e**) classical monocytes (CD14^+^CD16^−^) and in (**c**) and (**f**) inflammatory granulocytes proinflammatory monocytes (CD14^dim^CD16^+^) are shown. *p < 0.05; **p < 0.01; ***p < 0.005. Scattered plot with medians and interquartile

Percentages of CD32, TLR-2 and CD11b positive classical CD14^+^CD16^−^ monocytes were decreased in neonates compared with controls. Neonatal CD14^+^CD16^−^ monocytes showed a significant decrease in CD62L positive cells and CD62L amount on cell surface. In anti-inflammatory CD14^+^CD16^+^ cells CD62L amount was reduced (Table 1). The decrease of HLA-DR positive cells in neonates as well as the decrease of HLA-DR amount was measured for all three monocyte subpopulations (Fig. 4).

## Discussion

The function of innate immune cells in neonates is known to be different from adults. These immune alterations might be part of a balanced system between prevention of hyperinflammation and defence of pathogens [6]. Therefore, the regulation also of innate immune cells subpopulation might be more sophisticated than thought before.

### Granulocytes

Three different granulocyte subpopulations, classical CD16^+^CD62L^+^, anti-inflammatory CD16^+^CD62L^−^ and proinflammatory CD16^dim^CD62L^+^, were identified in adult blood [8] (Fig. 1a–c). In neonates, this study is the first to show that classical granulocytes are downregulated, while proinflammatory granulocytes are enhanced in neonates compared with adults*.*

Percentage of TLR-2 was enhanced on all granulocytes and their subpopulations while the TLR-2 expression was only reduced on proinflammatory granulocytes (Fig. 2e–g). TLR-2 recognizes a large number of ligands, especially on gram-positive bacteria [17, 18]. Apart from the well-characterized TLR-2-mediated inflammation, data exist supporting the notion that TLR-2 signalling can lead to the production of the anti-inflammatory cytokine Interleukin 10 [19, 20]. Therefore, differently regulated TLR-2 might be partly responsible for the diminished inflammatory neonatal immune response. Of note, expression of soluble factors in cord blood impairs TLR-4-mediated IL-12p70 production and enhances TLR-4-mediated IL-10 production over the first weeks of life [21].

Although percentages of CD11b and CD32 were downregulated on granulocytes in general, we could not detect these alterations in granulocyte subpopulations (Table 1). The reduction of CD11b in neonates is in line with the finding that a downregulation of CR3 complexes (CD11b/CD18) leads to an impaired recognition of gram-negative bacteria dependant on gestational age [12].

Our results indicate that neutrophils of term infants are partly diminished in their ROS production, especially in proinflammatory granulocytes (Fig. 4a–f). The reduced ROS production is in line with Usmani et al. [13]. Gessler et al. measured no difference in the percentage of neutrophils undergoing respiratory burst after being stimulated by fMLP or *E. coli* [14]. Similarly, we did not observe any alterations for granulocytes in general stimulated by fMLP and *E. coli* in ROS production. Nevertheless, granulocyte subpopulations showed an enhanced ROS production per cell especially after *E. coli* stimulation. Also Shigeoka et al. reported an enhanced ROS response, especially in stressed neutrophils [16]. The different findings within these studies might be due to divergent experimental setups in choice of stimulants and ROS production readouts. In our study DHR conversion by superoxide radicals into rhodamine is quantified [22]. These radicals are generated by NADPH oxidase which quantitatively differs between neonates and adults [23]. The enhanced NAPDH oxidase activity per cell in contrast to the diminished ROS response of proinflammatory granulocytes might be an indicator of balance between neonatal deficits on the one hand and intact immune responses to fight pathogens effectively on the other hand.

### Monocytes

Our data showed an enhanced percentage of anti-inflammatory monocytes while classical monocytes were decreased compared with adults (Fig. 1e–g). Others did not find those differences [24–26]. This might be due to the different classification of monocyte subpopulations since Sohlberg et al. as well as Murphy et al. only distinguished into two major subpopulations, CD14^++^CD16^−^ and CD14^+^CD16^+^ cells. Wisgrill et al. analysed counts of cells while our data compare subpopulation percentages. Especially the CD14^+^CD16^+^ monocytes have anti-inflammatory properties by the secretion of Interleukin 10 [27]. In addition to the lower frequencies of classical monocytes which are confirmed by the study of Sharma et al. in our cohort, these alterations might protect the host from overshooting proinflammatory immune responses facing invading pathogens [28]. In contrast to Sharma et al. we show an increase of anti-inflammatory monocytes without an alteration of inflammatory monocytes, which might be due to differences in gestational age and number of analysed subjects.

We found reduced monocytic HLA-DR expression resembling result by Nguyen et al. and Wisgrill et al. [25, 29]. Our study can expand this knowledge, since our data show a reduced percentage of HLA-DR positive cells in all monocyte subpopulations (Fig. 4a–f). Especially the reduction of CD62L on classical and anti-inflammatory monocytes seems to be a unique neonatal finding in our data since data from adult blood monocytes show CD62L expression especially on these both cell subpopulations but not in proinflammatory monocytes (Table 1) [30, 31].

### Limitations

This study only considers the production of ROS in vitro, but not its effectiveness in the killing of pathogens. *E. coli* as bacterial stimulus of ROS production has been described to play a role in the induction of *Escherichia coli* early-onset sepsis [32] as an important cause of mortality and morbidity in neonates. However our data cannot specify whether *E. coli*–induced ROS productions influence the clinical outcome of children. Also, only a limited amount of stimuli could be applied. Since Droussou et al. show an impaired respiratory burst in neonates challenged by sepsis, clinical or experimental ‘stress’ [33], the exact connection between differently regulated monocytes and granulocyte subpopulation, their activation state and the risk of infection still has to be revealed.

Our experiments analysed relative changes of immune cell subpopulations in neonates in comparison with healthy adults, but not absolute counts. Since we cannot provide any tendency for the influence of gestational age, birth weight and the incidence of infection, larger cohort particularly including preterm infants is needed.

## Conclusion

Although granulocyte and monocytes have been characterized by other marker (like CD66b, CD54 or CD49d), our study has demonstrated for the first time that subpopulations of granulocytes and monocytes can be defined by CD14, CD16 and CD62L in neonates. Furthermore, these subpopulations are differently regulated in term neonates compared with adults. Enhanced anti-inflammatory monocytes combined with reduced classical monocytes and classical granulocytes might protect hosts from overshooting immune responses. Although the neonatal is different from the adult immune regulation, our findings show that alterations do not include clear immune deficits. Therefore, it would be conceivable that overwhelming immune responses as well as a higher susceptibility towards infections are limited in neonates. Our data indicate that granulocyte and monocyte subsets, production of ROS as well as the regulation of activation marker are part of a balanced immune system in early life. Whether these balanced immune alterations increase the susceptibility of infections in preterm infants should be object of future studies.

## Supplementary information

**Additional file 1:** **Table S1.** Characteristics of analysed populations for activation and burst measurements in comparison to healthy young adults. Values presented as mean ± SD

**Additional file 2:** **Figure S1.** A/B Gating strategy for granulocyte and monocyte subpopulations and their activation marker: Representative probe of a new-born infant for activation marker on granulocyte and monocyte subpopulation. After single cell gating and determination of living cells by ZOMBIE, cells were gated by SSC-A and subpopulation marker (CD14, CD16, CD62L) in their subpopulation according to FMOs. CD14^dim^ monocytes and CD16^dim^ neutrophil population was distinguished by gating the 25th percentile of main neutrophil population.

**Additional file 3:** **Figure S2.** Gating strategy for oxidative burst setup***.*** Representative probe of a new-born infant to measure oxidative burst. To clearly distinguish monocyte and granulocyte subpopulations FMOs for anti-CD14, −CD16 and -CD62L gating was used. Subsets were defined as already published by Pillay et al. (2012). CD16^dim^ neutrophil population was distinguished by gating the 25th percentile of main neutrophil population

## Data Availability

The datasets acquired during and/or analysed during the current study are available from the corresponding author upon reasonable request.

## Abbreviations

DHR: Dihydrorhodamine 123
EDTA: Ethylene diamine tetraacetic acid
fMLP: N-formyl-methionyl-leucyl-phenylalanine
FMO: Fluorescence minus one
MFI: Mean fluorescence intensity
PFA: Paraformaldehyde
PMA: Phorbol 12-myristate 13-acetate
ROS: Reactive oxygen species
TLR: Toll-like receptor
