# Supplementary material for: Functional analysis of granulocyte and monocyte subpopulations in neonates
Source: Mol Cell Pediatr. 2019 Nov 28;6:5. doi: 10.1186/s40348-019-0092-y (PMC6884604; doi:10.1186/s40348-019-0092-y)
Supplement: Supplementary file 2 — Additional file 2: Figure S1. A/B Gating strategy for granulocyte and monocyte subpopulations and their activation marker: Representative probe of a new-born infant for activation marker on granulocyte and monocyte subpopulation. After single cell gating and determination of living cells by ZOMBIE, cells were gated by SSC-A and subpopulation marker (CD14, CD16, CD62L) in their subpopulation according to FMOs. CD14dim monocytes and CD16dim neutrophil population was distinguished by gating the 25th percentile of main neutrophil population. [file 40348_2019_92_MOESM2_ESM.zip › Suppl. Figure 1B.pdf]

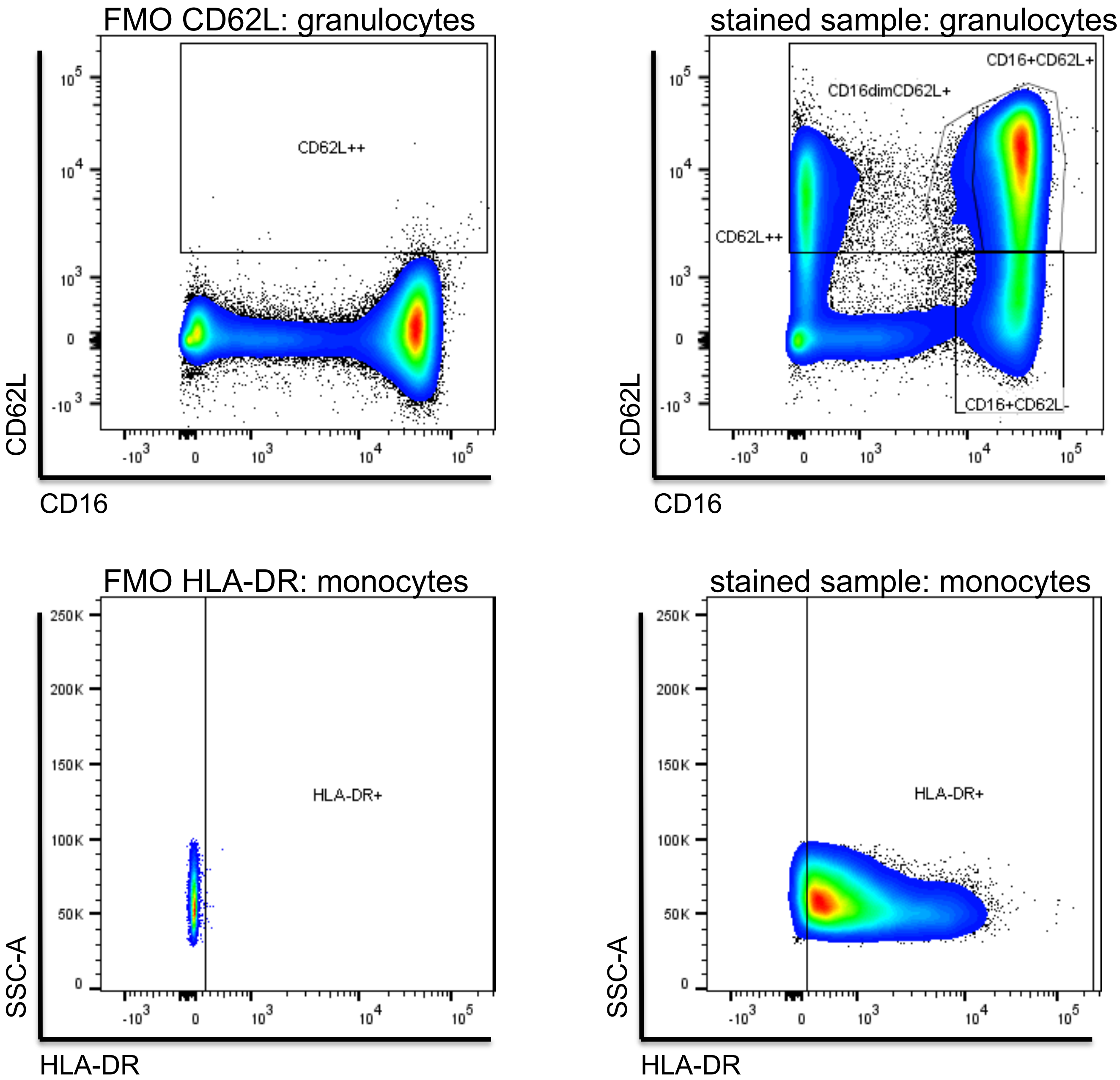
FMO CD62L: granulocytes
CD62L++
CD62L
CD16
stained sample: granulocytes
CD16+CD62L+
CD16dimCD62L+
CD62L++
CD16+CD62L-
CD62L
CD16
FMO HLA-DR: monocytes
HLA-DR+
SSC-A
HLA-DR
stained sample: monocytes
HLA-DR+
SSC-A
HLA-DR
