# Supplementary figures and images for: Functional analysis of granulocyte and monocyte subpopulations in neonates
Source: Mol Cell Pediatr. 2019 Nov 28;6:5. doi: 10.1186/s40348-019-0092-y (PMC6884604; doi:10.1186/s40348-019-0092-y)

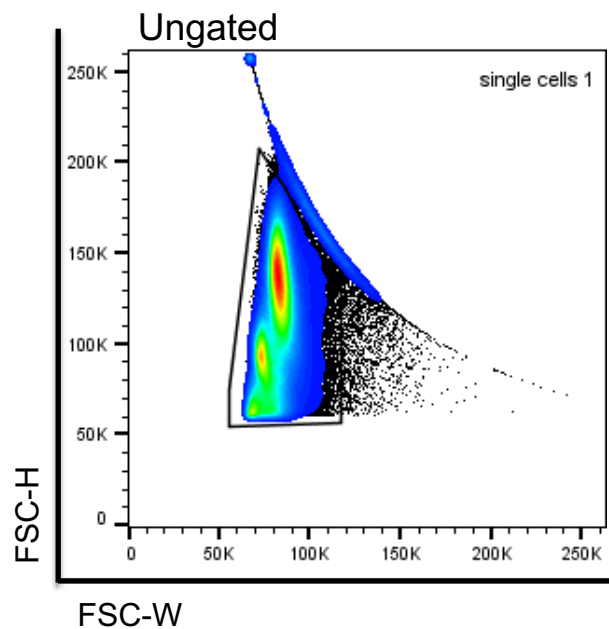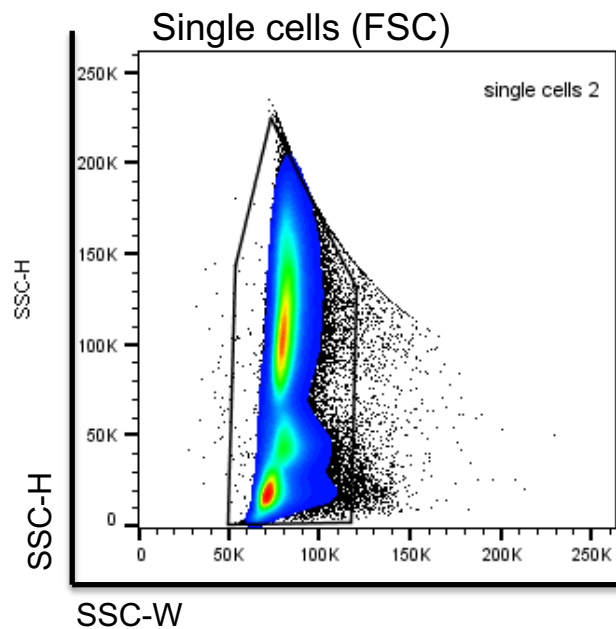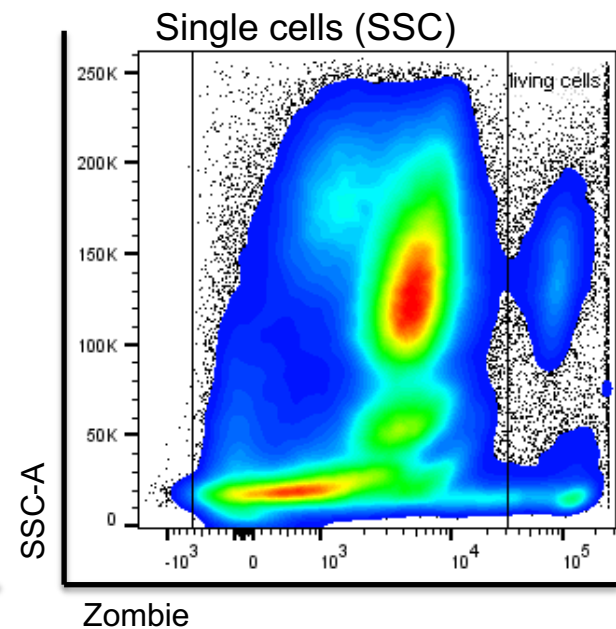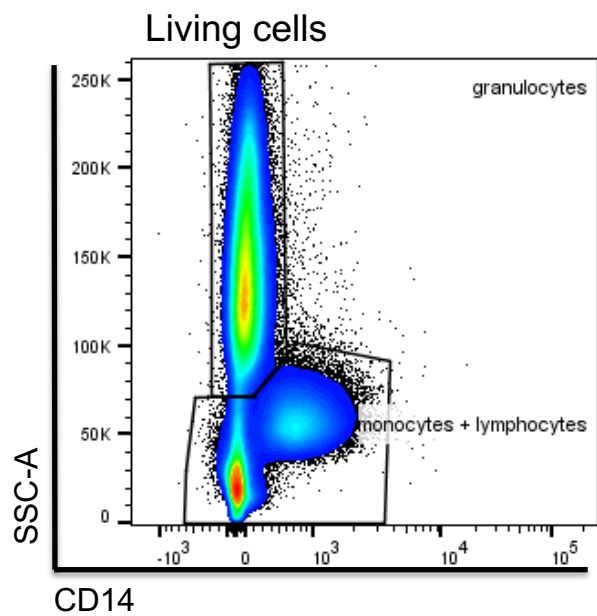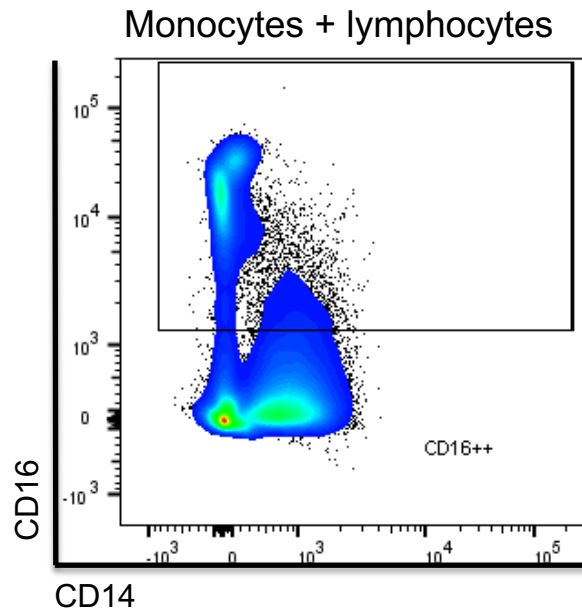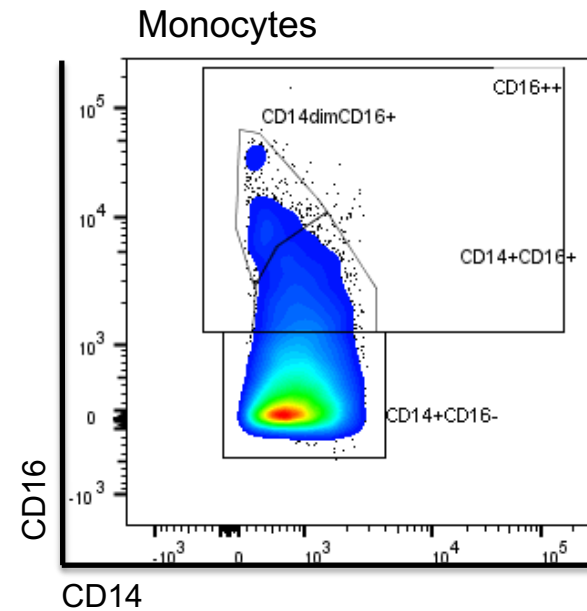

Supplement: Supplementary file 2 — Additional file 2: Figure S1. A/B Gating strategy for granulocyte and monocyte subpopulations and their activation marker: Representative probe of a new-born infant for activation marker on granulocyte and monocyte subpopulation. After single cell gating and determination of living cells by ZOMBIE, cells were gated by SSC-A and subpopulation marker (CD14, CD16, CD62L) in their subpopulation according to FMOs. CD14dim monocytes and CD16dim neutrophil population was distinguished by gating the 25th percentile of main neutrophil population. [file 40348_2019_92_MOESM2_ESM.zip › Suppl. Figure 1A.pdf]

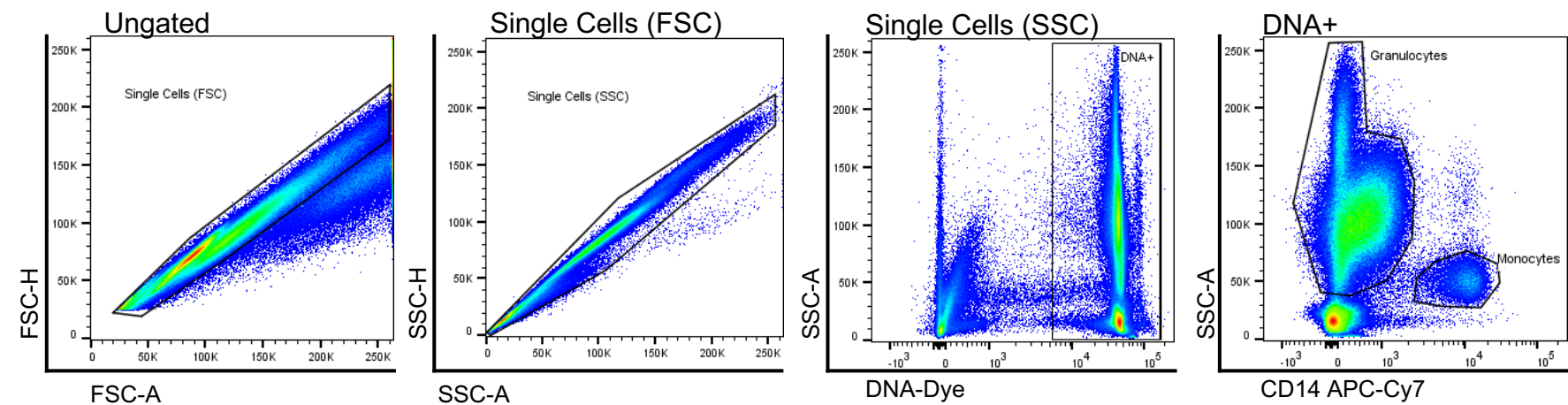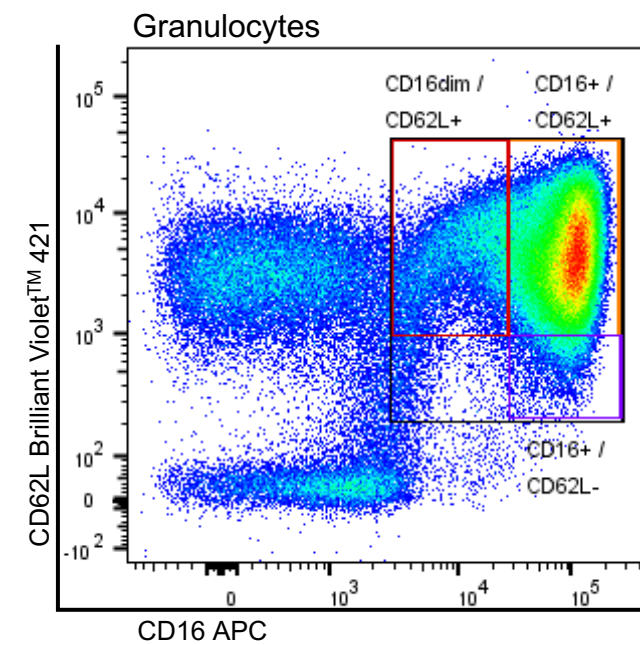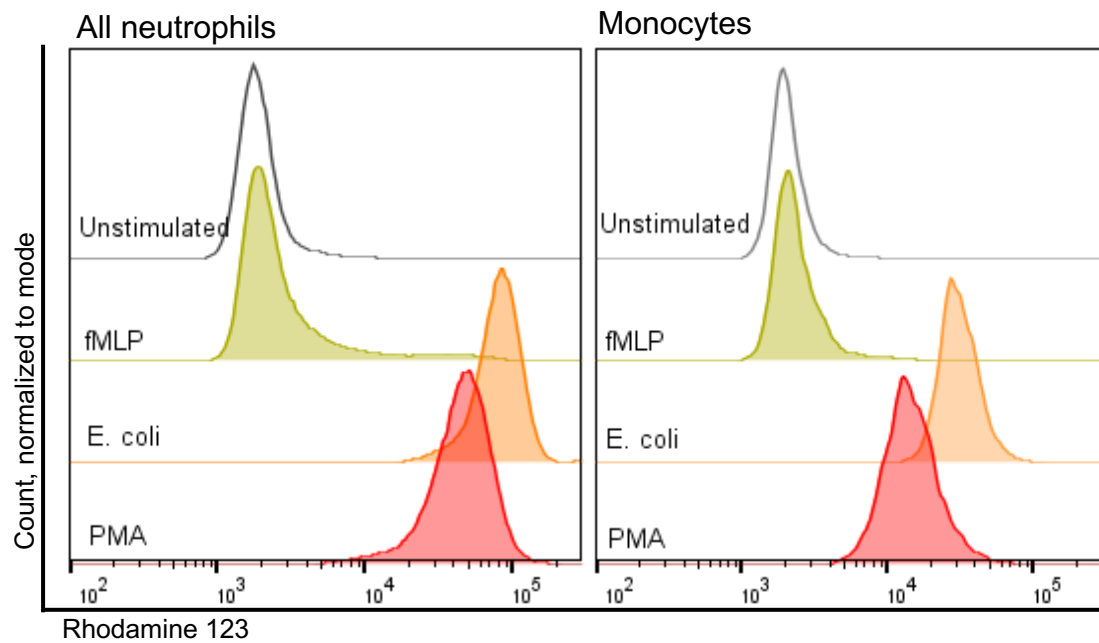

Supplement: Supplementary file 3 — Additional file 3: Figure S2. Gating strategy for oxidative burst setup. Representative probe of a new-born infant to measure oxidative burst. To clearly distinguish monocyte and granulocyte subpopulations FMOs for anti-CD14, −CD16 and -CD62L gating was used. Subsets were defined as already published by Pillay et al. (2012). CD16dim neutrophil population was distinguished by gating the 25th percentile of main neutrophil population [file 40348_2019_92_MOESM3_ESM.pdf]
